# Genetics of Cryptic Speciation within an Arctic Mustard, *Draba nivalis*

**DOI:** 10.1371/journal.pone.0093834

**Published:** 2014-04-01

**Authors:** A. Lovisa S. Gustafsson, Inger Skrede, Heather C. Rowe, Galina Gussarova, Liv Borgen, Loren H. Rieseberg, Christian Brochmann, Christian Parisod

**Affiliations:** 1 National Centre for Biosystematics, Natural History Museum, University of Oslo, Oslo, Norway; 2 Microbial Evolution Research Group (MERG), Department of Biosciences, University of Oslo, Oslo, Norway; 3 Botany Department, University of British Columbia, Vancouver, Canada; 4 Botany Department, Faculty of Biology and Soil Science, St Petersburg State University, St Petersburg, Russia; 5 Laboratory of Evolutionary Botany, Institute of Biology, University of Neuchâtel, Neuchâtel, Switzerland; University of Uppsala, Sweden

## Abstract

Crossing experiments indicate that hybrid sterility barriers frequently have developed within diploid, circumpolar plant species of the genus *Draba*. To gain insight into the rapid evolution of postzygotic reproductive isolation in this system, we augmented the linkage map of one of these species, *D. nivalis*, and searched for quantitative trait loci (QTLs) associated with reproductive isolation. The map adds 63 new dominant markers to a previously published dataset of 31 co-dominant microsatellites. These markers include 52 amplified fragment length polymorphisms (AFLPs) and 11 sequence-specific amplified polymorphisms (SSAPs) based on retrotransposon sequence. 22 markers displaying transmission ratio distortion were further included in the map. We resolved eight linkage groups with a total map length of 894 cM. Significant genotype-trait associations, or quantitative trait loci (QTL), were detected for reproductive phenotypes including pollen fertility (4 QTLs), seed set (3 QTLs), flowering time (3 QTLs) and number of flowers (4 QTLs). Observed patterns of inheritance were consistent with the influence of both nuclear-nuclear interactions and chromosomal changes on these traits. All seed set QTLs and one pollen fertility QTL displayed underdominant effects suggestive of the involvement of chromosomal rearrangements in hybrid sterility. Interestingly, *D. nivalis* is predominantly self-fertilizing, which may facilitate the establishment of underdominant loci and contribute to reproductive isolation.

## Introduction

The evolutionary mechanisms underlying the origin and build up of reproductive isolation (RI) continue to be the subject of considerable discussion and debate [1]-[3]. Recent inquiry into the processes leading to speciation in the presence of substantial gene flow suggests that divergent selection by the external environment can act as a central driver of speciation [4], [5]. Alternative, non-mutually exclusive processes involving other forms of selection or the action of forces other than selection may also be operating and deserve attention [6], [7]. The study of rapid speciation in selfing homoploid lineages may provide complementary insights into the processes underlying the rise of RI.

Multiple reproductive barriers typically isolate plant species [8]. Studies of the genetic basis of reproductive isolation offer valuable clues for evaluating the relative importance of the evolutionary mechanisms contributing to speciation [8]–[10]. Intrinsic postzygotic barriers can include: (i) cytonuclear incompatibilities that result from dysfunctional interactions between nuclear and cytoplasmic factors [11], creating asymmetric RI [12], [13]; (ii) Bateson-Dobzhansky-Muller (BDM) incompatibilities that result from the fixation of alternative alleles at one or several nuclear loci between lineages, leading to dysfunction when combined in hybrids [14], [15]; and (iii) structural divergence that results from the fixation of chromosomal rearrangements, reducing gene exchange between lineages by interfering with meiosis or reducing the level of recombination [16]–[19].

In theory, all these types of incompatibilities can accumulate in the genome with or without the action of selection. While genes underlying BDM type of incompatibilities in animals often exhibit the signature of positive selection [20], such evidence is surprisingly scarce in plants. However, several plant genes implicated in BDM incompatibility belong to gene families that are known to be targets of diversifying selection, and the development of cytonuclear incompatibilities often involves genetic conflict, which also implies a key role for selection [21]. As the neutral fixation of chromosomal rearrangements is considered unlikely to effectively drive RI [22], [23], BDM incompatibilities have been assumed to predominate over chromosomal rearrangements in the origin of intrinsic postzygotic isolation [24]. However, recent theoretical developments have highlighted selection in heterogeneous environments as an efficient promoter of the establishment of chromosomal rearrangements, suggesting that genome restructuring may play a significant role in the rise of new species [18], [25], [26].

Previous intraspecific crossing experiments revealed a surprisingly high frequency of recent (presumably Pleistocene) cryptic speciation events in three circumpolar, diploid and predominantly self-fertilizing *Draba* species [27]. To analyze quantitative trait loci (QTLs) underlying intrinsic postzygotic RI in one of these species, *D. nivalis*, Skrede et al. [28] recently produced a linkage map based on 50 microsatellites. Subsequent analysis of reproductive phenotypes in a segregated population suggested that multiple genetic mechanisms, including possible chromosomal rearrangements, might account for the rapid evolution of RI in this system. Under-dominant loci, most probably due to microchromosomal rearrangements, were associated with seed fertility, whereas nuclear and possibly cytonuclear incompatibilities were associated with pollen fertility. Here, we gain insight into the genetic mechanisms underlying RI in *D. nivalis* and further assess the impact of underdominant loci as well as nuclear-nuclear, and cyto-nuclear incompatibilities. The existing genetic map [28] was augmented by the addition of amplified fragment length polymorphisms (AFLPs) and sequence-specific amplified polymorphisms of retrotransposon insertions (SSAPs). A genetic linkage map containing eight linkage groups (*D. nivalis* 2*n* = 16) was inferred from analysis of 99 codominant and dominant markers. QTL analysis detected fourteen QTLs in total, including four QTLs associated with pollen fertility, three with seed set, three with flowering time and four with number of flowers.

## Materials and Methods

### Mapping population and previous data sets

We used the mapping population and the following material and data sets produced by Skrede et al. [28]. The mapping population comprised 382 F_2_ individuals of *D. nivalis* derived from self-pollination of a semi-fertile F_1_ hybrid obtained from a cross performed by Grundt et al. [27] between a *D. nivalis* plant from Alaska (008-7; maternal parent) and a *D. nivalis* plant from Norway (045-5; paternal parent; see [27] for locality information). To compare traits, Skrede et al. [28] raised nine additional F_1_ hybrids, seven maternal plants, and one paternal plant to maturity under the same conditions as the F_2_ population. The following traits were extracted from Skrede et al. [28]: (*i*) seed set (i.e. average number of seed per fruit following spontaneous self-fertilization), (*ii*) pollen fertility (i.e. % of fully stained pollen grains after staining with cotton blue), (*iii*) flowering time (i.e. number of days from the opening of the first flower on the first flowering plant) and (*iv*) number of flowers (see details in [28]). Number of flowers was square root transformed to obtain a normal distribution, whereas the other traits did not statistically deviate from a normal distribution. DNA was extracted from fresh leaf material following the plate extraction protocol of Qiagen (Valencia, CA) and all plants were genotyped at 50 microsatellite loci [28].

### AFLP and SSAP genotyping

We genotyped the mapping population using AFLP [29] and SSAP [30] markers. Both types of markers are based on the digestion of genomic DNA with restriction enzymes and ligation of adapters to generate fragments to be amplified by PCR, but SSAP uses a transposable element (TE)-derived primer to amplify fragments encompassing the border of TE insertions and flanking genomic regions up to the adaptor [31].

Three TEs showing evidence of recent transpositional activity in Brassicaceae, TRIM-Br, SB2, and AtC10, were investigated here. TRIM-Br is a 350 bp Terminal Repeat In Miniature retrotransposon identified in *Brassica rapa* and *B. oleracea* [32]. Polymorphic insertions within *B. rapa* suggest that TRIM-Br has been active during domestication and possibly involved in the evolution of new chimeric genes [33]. SB2 (formerly known as RathE1 or AtSN2) is a 150 bp Short Interspersed Nuclear Element (SINE) reported in *Arabidopsis thaliana* and *B. oleracea* [34], [35]. Nearly 65% of SB2 insertions in *A. thaliana* are intercalated with genes [36]. AtC10 is a 5000 bp *copia* long terminal repeat (LTR) retrotransposon described from several insertions as well as expressed sequence tags (ESTs) suggestive of recent activity in *A. thaliana* [37].

SSAP fingerprints for TRIM-Br were generated with the FAM-labelled primer Br1&2-DP (5′-GCTTCCAACYYAAAACCAATTGG-3′; [33]. For SB2, insertions from *A. thaliana* and *B. oleracea* were aligned using ClustalW and a SSAP, VIC-labelled primer SB2 (5′-AAACTAATATTACATGTTGTGGTTTCG-3′) was designed across a conserved region. For AtC10, a recent insertion of *A. thaliana* (AC007259.1) was blasted (blastn) against *B. rapa* to identify recent insertions (KBrH013M23, KBrB042O03). Insertions were aligned using ClustalW and the SSAP NED-labelled primer AtC10 (5′-TCAAACACTTAAACACTTTCTCCAT-3′) was designed from conserved regions at 30 bp from the 5′-end of the LTR.

We tested 72 AFLP and 36 SSAP primer combinations on eight individuals (i.e. the two parents, three F_1_ hybrids and three F_2_ hybrids) to identify loci that were polymorphic among the parents, present in the F_1_ hybrids, and segregating among F_2_ hybrids. Unfortunately, few polymorphic loci were found between the parents. The complete mapping population was genotyped with 13 AFLP and seven SSAP primer combinations (Table S1). Both AFLP and SSAP procedures were performed following Parisod and Christin [38]. For AFLP primer combinations, 2 μL 6-FAM, 2 μL VIC and 3 μL NED labelled selective PCR products from each individual were mixed, whereas equal volumes of SSAP PCR products were mixed. Mixed PCR products were analysed on an ABI 3730XL capillary sequencer (Applied Biosystems, Foster City, CA, USA; service provided by Macrogen Inc. Seoul, Korea). Loci were scored from raw data as present/absent using GeneMapper version 3.7 (Applied Biosystems).

### Linkage analysis

We tested each marker for transmission ratio distortion (TRD) using a chi-square goodness of fit test within AntMap [39], expecting a 1:2:1 relationship for codominant microsatellite markers and a 1:3 relationship for dominant AFLP/SSAP markers. Distorted markers disrupted the linkage mapping, therefore co-dominant markers having <15% of homozygotes and dominant markers being present/absent in <15% or >40% individuals of the mapping population were initially excluded from the map construction. These TRD markers were then assigned tentative positions on the linkage map by sequential addition (see below). The absolute deviation of the parental homozygote frequency from the Mendelian expectation of 0.25 was further estimated for each of the mapped TRD markers. Parental transmission bias was thus evaluated through one estimate for each dominant marker (i.e. AA/BB excess or deficit) and two semi-independent values (i.e. AA excess/BB deficit or BB excess/AA deficit) for co-dominant markers.

The genetic map was constructed using R/qtl [40], following [41]. Individuals and markers with >20% missing data were excluded. Linkage groups were formed using the non-distorted markers by pairwise consideration of estimated recombination fraction and minimum LOD score, i.e. two markers were placed in the same linkage group if the estimated recombination fraction was ≤ 0.35 and LOD score ≥ 5. Linkage groups with less than three markers were excluded. Marker order in the linkage groups was estimated by picking an arbitrary pair of markers and then randomly adding an additional marker, one at a time, in the best supported position. Alternative marker orders were tested by considering all possible orders of markers in a window of four, while keeping a fixed order for markers outside the window. We retained the marker order associated with a maximized likelihood score (error probability 0.01) and minimized number of crossover events. Excluding one marker at a time and recording the difference in map length and LOD score showed that removal of no single marker was justified by a significant decrease in map length or number of crossover events. After completion of this initial map, the distorted markers were added to the map one at a time using R/qtl.

### QTL analyses

Composite interval mapping of four phenotypic traits (i.e. pollen fertility, seed set, flowering time and number of flowers) was performed in R/qtl [40], using default settings. Hidden Markov models (HMMs) estimated QTL genotype probabilities at a 1 cM density as a function of the genotypes at the nearest markers, assuming no crossover interference (i.e. genotypes of dominant markers were inferred using the information from the codominant markers). Phenotypes were then mapped using the Haley Knott regression with a genome-wide LOD significance threshold for each trait assessed with 1000 permutations (alpha  =  0.05).

QTLs with very high LOD scores may hinder detection of additional loci affecting the trait of interest. Therefore, QTLs with a LOD >2 were sequentially omitted (i.e. “drop one QTL at a time” analysis) to compare the LOD of such reduced models to the LOD of the full model. Significance of this test was assessed by both the chi-squared distribution of LOD scores and the *F*-statistics of the ratio of the squared residuals. Interacting QTLs were further checked with the same approach. The linkage map with QTLs was displayed using MapChart [42].

## Results

**Figure 1:**
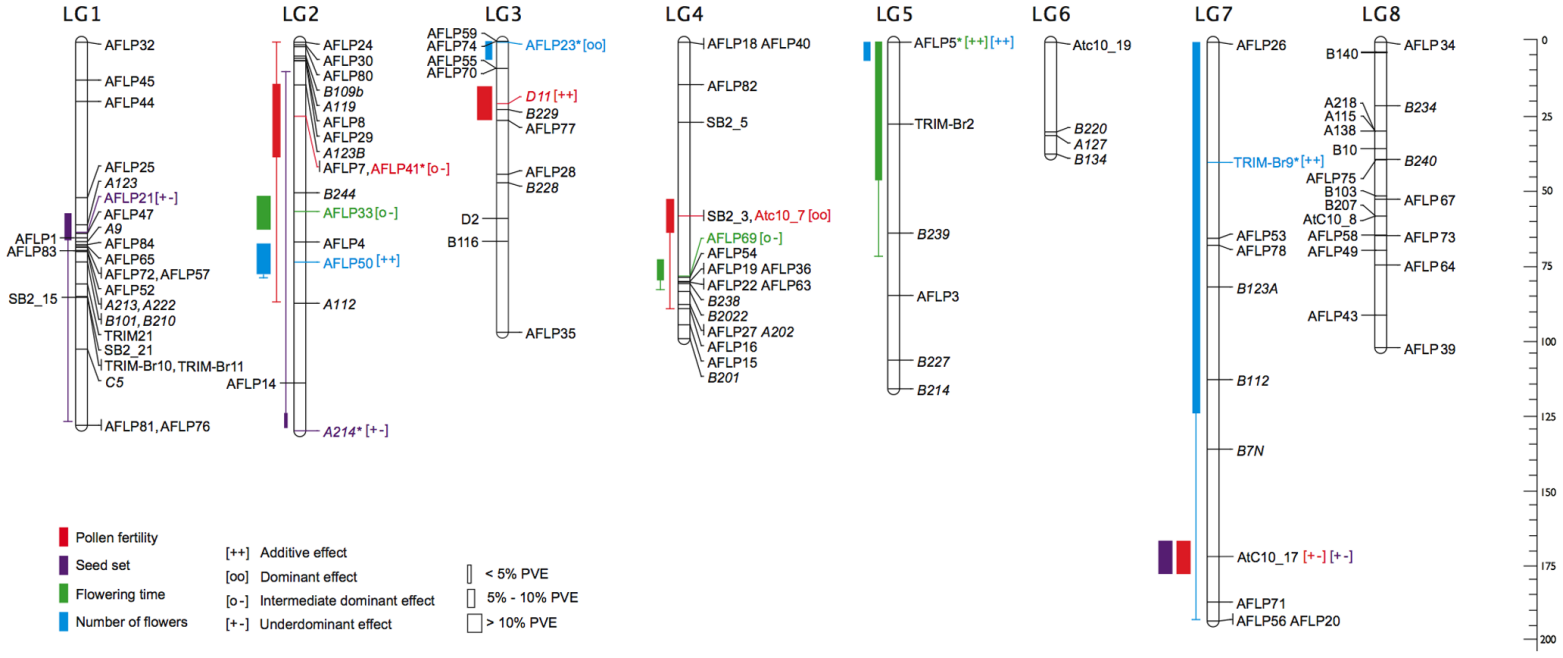
Genetic linkage and QTL mapping of *Draba nivalis*. Linkage map of the *D. nivalis* genome with eight linkage groups (LG1–LG8) and quantitative trait loci (QTLs). Scale indicates distance in centiMorgans (cM). Markers presented on the right of each linkage group represent the non-distorted markers on which the map construction and QTL analysis was employed. Subsequently included TRD markers (i.e. co-dominant markers having <15% of homozygotes and dominant markers being present/absent in <15% or >40% individuals) are displayed on the left of each LG. QTL bars, colored as mentioned in the panel, indicate LOD1 intervals. The lines extending from QTL bars indicate LOD2 intervals, and the thickness of QTL bars indicates Percent Phenotypic Variation Explained (PVE). Colored markers indicate the marker closest to the LOD peak for that particular QTL and the corresponding effect is indicated as either additive [++], dominant [oo], intermediate dominant [o −] or underdominant [+ −]. QTLs uncovered after the effect of other QTLs were accounted for are indicated with an asterisk. Total map length is 894 cM.

**Figure 2:**
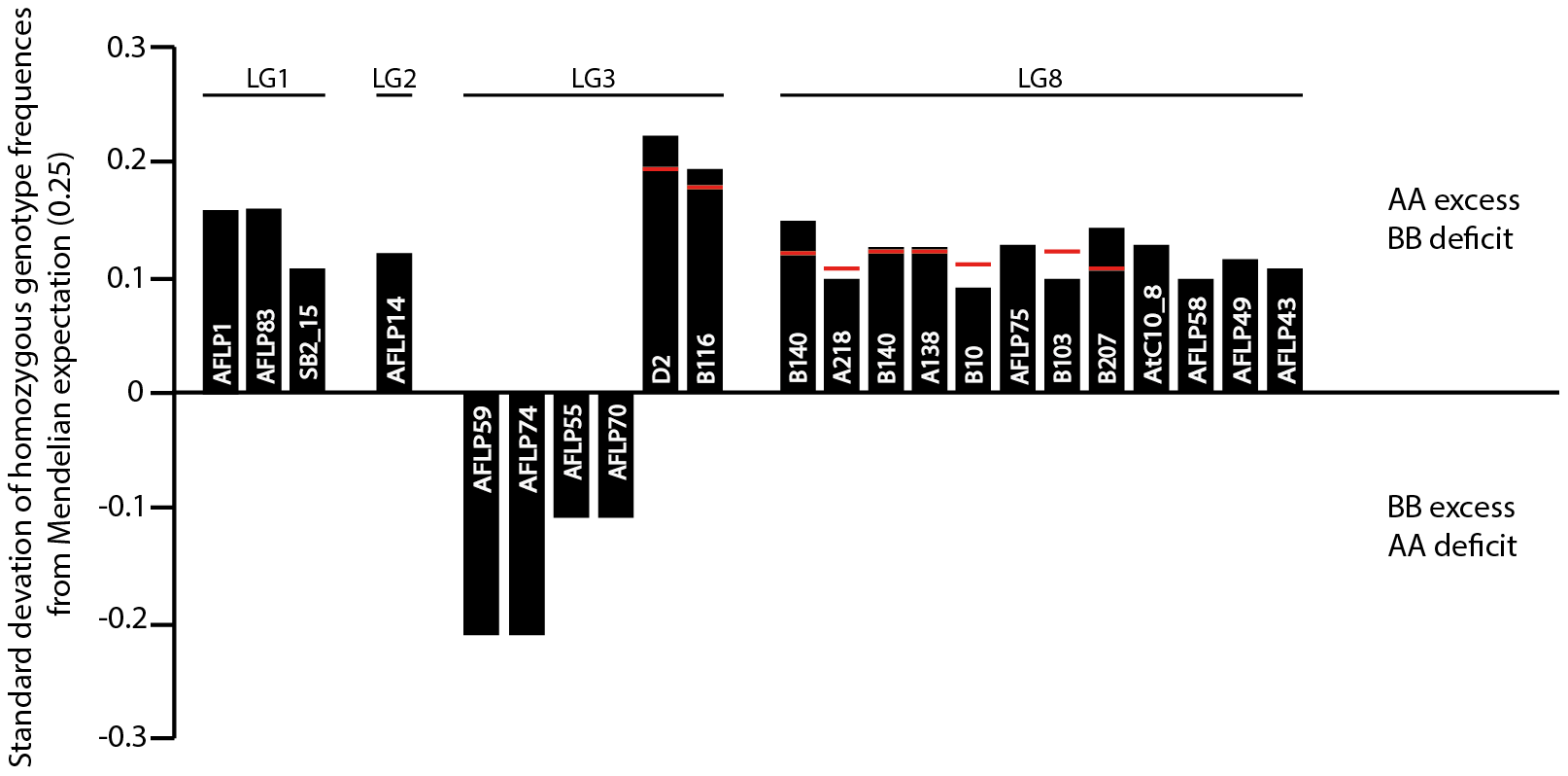
Transmission ratio distortion across the *Draba nivalis* linkage map. Histograms indicate the direction and magnitude of deviation of the parental homozygote frequency from the Mendelian expectation of 0.25 as positive in case of AA (male) excess and BB (female) deficit, or negative in case of AA deficit and BB excess. For dominant markers, either the AA or BB excess / deficit is indicated by the black bars. For co-dominant markers, the black bar indicates the AA excess, whereas the red lines indicate the BB deficit.

We analyzed 128 loci satisfying our criteria in 359 F_2_ individuals. Twenty-nine distorted markers (22.6%) were initially omitted from map construction. A linkage map was generated from the 99 remaining markers, and three linkage groups containing < three markers each were excluded from further analyses. Thus, the map employed for QTL analyses and shown in Fig. 1 included 94 markers (31 microsatellites, 52 AFLP and 11 SSAP), forming eight linkage groups (LG1 to LG8) that presumably correspond to the eight chromosomes of *D. nivalis*. Total map length was 894 cM, with averages of 112 cM per linkage group (ranging from 37 to 192 cM) and 10 cM between markers. Subsequently added TRD markers (i.e. nine microsatellites, 11 AFLP, two SSAP) mapped to several linkage groups, with a noticeable bias towards LG3 and LG8 (Fig. 1, Table S1). The absolute deviation of the parental homozygote frequency from Mendelian expectations showed a bias towards the paternal alleles (Fig. 2).

### QTL results

A total of 14 significant QTLs were detected, of which four were associated with pollen fertility, three with seed set, three with flowering time and four with number of flowers (Fig. 1; Table 1)

**Table 1:** QTL mapping of *Draba nivalis*.

Trait	LOD threshold (alpha = 0.05)	Marker closest to LOD peak	LG	Position (cM)	LOD score 1	LOD score 2	LOD1- interval	LOD2 -interval	PVE (%)	Ratio	Mean AA ± SD	Mean AB ± SD	Mean BB ± SD
Pollen fertility	3.64	AFLP41	2	24	2.63	7.05	14–38	0–86	6.77	72:235:52	62.36 ± 21.66	52.76 ± 22.49	49.18 ± 19.59
		D11	3	19	7.12	10.47	15–25.9	15–25.9	10.47	70:184:102	41.87 ± 20.73	54.72 ± 20.90	61.49 ± 22.47
		AtC10_7	4	62	4.25	7.95	52–63	52–88	7.68	72:235:52	44.71 ± 19.12	56.74 ± 22.82	56.64 ± 21.75
		AtC10_17	7	166	6.65	12.07	165–176	165–176	11.99	108:193:58	60.89 ± 20.57	49.86 ± 21.81	55.89 ± 23.94
Seed set	3.57	AFLP21	1	62.9	3.73	6.63	57–66	57–126	7.66	138:157:64	9.25 ± 3.60	7.75 ± 3.59	8.27 ± 3.62
		A214	2	128.6	2.9	4	123–128	10–128	4	97:197:63	8.63 ± 3.67	7.99 ± 3.59	9.38 ± 3.67
		AtC10_17	7	173	8.46	10.83	165–176	165–176	12.88	110:188:61	9.63 ± 3.62	7.55 ± 3.40	8.76 ± 3.78
Flowering time	3.54	AFLP33	2	55	11.38	13.69	51–62	51–62	14.72	59:208:92	16.83 ± 3.36	17.69 ± 4.29	21.41 ± 5.66
		AFLP69	4	73	5.46	8.47	72–79	72–82	8.79	81:189:89	20.69 ± 5.07	18.25 ± 4.84	17.14 ± 4.11
		AFLP5	5	11	3.12	4.97	0–46	0–71	5.04	139:143:77	19.68 ± 4.98	17.50 ± 4.64	18.29 ± 4.70
Number of flowers	3.59	AFLP50	2	72	9.79	13.03	67–77	67–78	13.66	78:175:106	4.30 ± 2.43	3.18 ± 1.82	2.32 ± 1.60
		AFLP23	3	3	3.28	5.89	0–6	0–6	5.88	74:185:100	2.44 ± 1.83	3.48 ± 2.12	3.14 ± 1.87
		AFLP5	5	5	4.19	5.74	0–6	0–6	5.73	141:139:79	2.70 ± 1.84	3.40 ± 2.02	3.60 ± 2.21
		TRIM-Br_9	7	21	2.15	5.79	0–123	0–191	5.78	82:182:95	2.97 ± 1.83	3.06 ± 1.97	3.57 ± 2.28

Results from QTL mapping of the F_2_ population of *Draba nivalis*, indicating the 95% significance threshold value for each trait. The marker closest to the LOD score peak for the particular QTL is indicated (LOD score 1 indicate the LOD peak in the initial analysis and LOD score 2 indicate the LOD peak after the effects of other QTLs were accounted for). Percent PhenotypicVariation Explained (PVE) is indicated. Ratio indicates the proportion of homozygotes for AA (paternal alleles), heterozygotes, and homozygotes for BB (maternal alleles). Mean value for each trait is indicated for homozygote F_2_ individuals for paternal alleles (AA), heterozygote F_2_ individuals (AB) and homozygote F_2_ individuals for maternal alleles (BB), with inferred genotypes in italics.

Pollen fertility QTLs exceeding the 95% significance threshold value of 3.64 LOD were detected on LG3, LG4 and LG7, whereas an additional QTL was uncovered on LG2 after the effects of other QTLs were accounted for in the model (Table 1). There was considerable variation in gene action among the pollen fertility QTLs (as assessed based on the marker closest to the LOD peak). This includes intermediate dominance on LG2 (marker AFLP41), additivity on LG3 (marker D11), dominance on LG4 (marker AtC10_7), and underdominance on LG7 (marker AtC10_17). The maternal allele was dominant to the paternal allele for the QTLs on LG2 and LG4. Pollen fertility QTLs on LG3 and LG7 showed evidence of epistasis (LOD  =  2.487, p  =  0.022; F  =  2.635, p  =  0.034).

Seed set QTLs exceeding the 95% significance threshold value of 3.57 LOD were detected on LG1 and LG7, whereas an additional QTL was uncovered on LG2 after the effects of other QTLs were accounted for in the model (Table 1). All three QTLs were underdominant (marker closest to LOD peak on LG1, AFLP21; LG2, A214; LG7, AtC10_17). No significant evidence of epistasis among QTLs was detected.

Flowering time QTLs exceeding the 95% significance threshold value of 3.54 LOD were detected on LG2 and LG4, whereas an additional QTL was uncovered on LG5 after the effects of other QTLs were accounted for in the model (Table 1). The QTLs on LG2 and LG4 had intermediate dominance effects (marker closest to LOD peak: AFLP33 and AFLP69, respectively), whereas the QTL on LG5 displayed additive gene action (marker closest to LOD peak: AFLP5). Flowering time QTLs on LG2 and LG4 showed evidence of epistasis (LOD  =  2.613, p  =  0.017; F  =  2.608, p  =  0.022).

Number of flower QTLs exceeding the 95% significance threshold value of 3.59 LOD were detected on LG2 and LG5, whereas two additional QTLs were uncovered on LG3 and LG7 after the effects of other QTLs were accounted for in the model (Table 1). The QTLs on LG2, LG5, and LG7 had additive effects, whereas for the LG3 QTL, the paternal allele was dominant to the maternal allele. No significant evidence of epistasis among QTLs was detected.

In addition, we identified three TRD markers co-localized with seed set QTLs on LG1 and LG2, two TRD markers possibly co-localized with pollen QTL on LG3, and four TRD markers that potentially mapped to number of flowers QTL on LG3 (Table S2).

## Discussion

The 894 cM genetic map presented here is based on robust linkage analysis of 128 codominant and dominant markers, resolving eight linkage groups, possibly corresponding to the eight chromosomes of *D. nivalis*. The map distances reported are based on 94 well-behaved markers, after having initially excluded TRD markers and three very small linkages. The vast majority of markers with subsequently included TRD, except those on LG3, demonstrate a significant bias towards excess of male alleles (or deficit of female alleles) in the F_2_ mapping population, and more than half mapped to LG8 (Table S1, Fig. 2). Such unidirectional transmission of specific chromosomal segments is commonly reported for both intra- and inter- specific crosses [43]–[45], an observation consistent with the action of biological processes rather than chance or technical errors. Inbreeding depression can be a major source of TRD in mapping populations, but is unlikely in the highly selfing *D. nivalis* because severely deleterious recessive alleles have likely been purged by natural selection [46]. Non-Mendelian genotypic ratios as observed here more likely arise from intergenomic interactions between parental genomes, resulting from (a) events during meiosis or early gametophyte development, (b) differential success of F_1_ gametes, (c) differential survival of F_2_ zygotes or (d) all of these mechanisms (see Fishman et al. [52] for a detailed account). The moderate male bias in genotype frequencies in our F_2_ population suggests that male alleles contributed by the male parent (045-5 from Norway) had a competitive advantage during F_1_ selfing. Although the clustering of TRD markers on LG8 are consistent with a system of drive, our data cannot offer conclusive identification of the mechanisms leading to male haplotype overrepresentation among F_2_ progeny.

QTL analyses of flowering time and number of flowers are consistent with the results of Skrede et al. [28]. Multiple QTLs were found for both traits and allelic effects ranged from additive to dominant. No evidence of underdominance was observed for these traits, suggesting a similar architecture to that typically reported for species differences that are not associated with intrinsic postzygotic barriers [15]. QTLs for both traits were detected at the same location on LG5 (close to AFLP5), suggesting pleiotropy or close linkage between causal genes. The early flowering QTL allele is correlated with a high number of flowers, consistent with response to environmental constraints such as the short season available for *D. nivalis* to reproduce in the Arctic [47]. While this study represents an early effort toward understanding the genetic basis of flowering time variation within *D. nivalis*, the impact on RI is likely to be minimal because QTL effects are small and parental lines show considerable overlap in flowering time. Instead, self-fertilization combined with effective geographic isolation [27], [48] appears to be largely responsible for prezygotic RI in *D. nivalis*.

The genetic basis of intrinsic postzygotic reproductive barriers appears to be different from that observed for flowering time and flower number. QTL patterns consistent with both nuclear-nuclear interactions and chromosomal changes were observed, corroborating the conclusion of Skrede et al. [28] that multiple genetic mechanisms account for the accumulation of hybrid incompatibilities within *D. nivalis*. Postzygotic RI was associated with four and three QTLs underlying pollen fertility and seed set, respectively. QTLs underlying pollen fertility displayed additive to dominant or underdominant effects, whereas all seed set QTLs exhibited underdominant effects. In contrast to Skrede et al. [28], and other studies suggesting the importance of cytonuclear incompatibilities in RI (e.g. [12]), we did not detect consistently higher fertility associated with maternal alleles for pollen QTLs. Maternal alleles were indeed associated with increased fertility in F_2_ hybrids for the QTL on LG3 (i.e. near D11) and LG4 (i.e. near AtC10_7), but the QTL on LG2 (i.e. near AFLP41) showed the opposite pattern and most likely represents a nuclear-nuclear BDM incompatibility. This is consistent with the hypothesized importance of BDM incompatibilities in the evolution of new species [8].

The higher fitness of some of the F_2_ hybrids as compared to the F_1_ hybrids (see [28] for details), as well as the mapping of underdominant QTLs for traits associated with seed set and pollen fertility, suggest involvement of chromosomal rearrangements in the origin of RI in this system. In particular, the underdominant seed set QTLs on LG7, LG1 and LG2 are consistent with multiple restructuring events among lineages, fostering RI in *D. nivalis* following chromosomal models of speciation. The influence of BDM incompatibilities affecting a single locus without loss of fitness in geographically isolated populations cannot be ruled out, but this scenario seems unlikely given the short time period since the populations of *D. nivalis* diverged (presumably Pleistocene) to accumulate recurrent mutations in the same gene [49]. The small effective population size of *D. nivalis* [56] may facilitate the fixation of chromosomal changes through genetic drift.

Only a few studies have recently examined the processes underlying the fixation of chromosomal rearrangements among plant lineages and their impact on RI. Adaptive QTLs underlying prezygotic isolation between *Mimulus lewisii* and *M. cardinalis* mapped to regions of suppressed recombination corresponding to reciprocal translocations and inversions [50]. In particular, underdominant male sterility was associated with two of the five rearrangements distinguishing the two species, suggesting that chromosomal restructuring was crucial for the build-up of RI. In contrast, no rearrangements unique to the self-pollinating species *M. parishii* were reported [50], suggesting that drift was not the primary process involved in fixation of rearrangements in that system. Inversions were indeed shown to have contributed to adaptation and multiple isolating barriers within *M. guttatus*, indicating that selection may drive the fixation of chromosomal rearrangements in some plant systems and thus lead to chromosomal speciation [51].

The two QTLs on LG7 (i.e. near AtC10_17) showing underdominant effects for both pollen fertility and seed set were associated with polymorphism at an insertion of the 5000 bp LTR retrotransposon AtC10. Plant genomes usually contain hundreds of such insertions [52], [53] that may map to QTL intervals without any significant effect on focal phenotypes. The association revealed here is not necessarily coincidental as retrotransposons have been shown to contribute significantly to genome evolution [54]-[56]. Polymorphic insertions may modify local recombination rates by disrupting colinearity and/or inducing formation of heterochromatin [57]-[59]. Accordingly, such small-scale chromosomal changes or the resulting linkage between previously segregating BDM loci would behave as an underdominant locus [60] without the requirement of strong genetic drift for fixation [17], [18]. Our evidence of chromosomal changes associated with TE dynamics, while indirect, calls for further investigation to reconcile chromosomal models of speciation with theoretical assumptions [61]. Sequencing of corresponding QTL intervals may help to shed light on the exact architecture of loci causing RI in *D. nivalis*.

A phylogeographic study of *D. nivalis* showed that the parents of our mapping population represent different genetic (AFLP) groups that probably diverged in distinct glacial refugia [49]. However, the number of genetic groups detected was insignificant compared with the numerous cryptic biological species highlighted by Grundt et al. [27], suggesting rapid development of sterility barriers. The incompatible loci affecting sterility may thus still be segregating, as shown in *Drosophila melanogaster* [62]. Accordingly, chromosomal rearrangements might support the consolidation of RI [63], but the relative contributions of drift and selection to speciation in *D. nivalis* remain undetermined. The predominantly selfing reproductive strategy of *D. nivalis* most likely contributed to the rapid build up of RI by reducing gene flow and effective recombination between populations, and given that adaptation occurs, possibly increasing the speed of fixation [64].

### Data availability

Data will be made available on Dryad upon publication

## Supporting Information

Table S1 **AFLP and SSAP primer combinations used, indicating number of polymorphic alleles present in each combination. Marker names are given.**
(DOCX)

Table S2 **Markers excluded from initial map construction because of transmission ratio distortion (TRD; co-dominant markers having <15% of homozygotes and dominant markers being present/absent in <15% or >40% individuals of the mapping population were omitted.** Information on likely placement in the *Draba nivalis* genome, association with QTL traits, proportion of TRD, and mean fertility of the F_2_ individuals associated with the particular marker.
(DOCX)
