# Supplementary material for: Genetics of Cryptic Speciation within an Arctic Mustard, Draba nivalis
Source: PLoS One. 2014 Apr 1;9(4):e93834. doi: 10.1371/journal.pone.0093834 (PMC3972243; doi:10.1371/journal.pone.0093834)
Supplement: Table S1 — AFLP and SSAP primer combinations used, indicating number of polymorphic alleles present in each combination. Marker names are given. (DOCX) [file pone.0093834.s001.docx]

**Supporting Information**

Table S1. AFLP and SSAP primer combinations used, indicating number of polymorphic alleles present in each combination. Marker names are given.

| EcoRI | MseI | Polymorphic alleles | Marker name | |
| --- | --- | --- | --- | --- |
| primer | primer |  | From | To |
|  |  |  |  |  |
| AFLP: |  |  |  |  |
| 6-FAM ATC | CAA | 11 | AFLP1 | AFLP11 |
| VIC AGA | CAC | 11 | AFLP12 | AFLP22 |
| NED AGT | CAT | 13 | AFLP23 | AFLP35 |
| 6-FAM ACC | CTC | 4 | AFLP36 | AFLP39 |
| VIC AAT | CAC | 7 | AFLP40 | AFLP46 |
| NED AGT | CTC | 7 | AFLP47 | AFLP53 |
| 6-FAM ATC | CTC | 6 | AFLP54 | AFLP59 |
| VIC AAT | CAA | 5 | AFLP60 | AFLP64 |
| NED AGT | CGT | 3 | AFLP65 | AFLP67 |
| 6-FAM ACA | CAT | 7 | AFLP68 | AFLP74 |
| VIC AAT | CTC | 4 | AFLP75 | AFLP78 |
| NED AAC | CTC | 3 | AFLP79 | AFLP91 |
| 6-FAM ATC | CAT | 3 | AFLP82 | AFLP84 |
|  |  |  |  |  |
| SSAP: |  |  |  |  |
| TRIM-Br | CGC | 2 | TRIM-Br1 | TRIM-Br2 |
| SB2 | CAA | 3 | SB2_3 | SB2_5 |
| AtC10 | CAC | 2 | AtC10_7 | AtC10_8 |
| TRIM | CTA | 4 | TRIM-Br9 | TRIM-Br12 |
| SB2 | CCA | 3 | SB2_13 | SB2_15 |
| AtC10 | CTC | 1 | AtC10_16 | - |
| AtC10 | CCA | 4 | AtC10_17 | AtC10_20 |
