# Supplementary material for: Genetics of Cryptic Speciation within an Arctic Mustard, Draba nivalis
Source: PLoS One. 2014 Apr 1;9(4):e93834. doi: 10.1371/journal.pone.0093834 (PMC3972243; doi:10.1371/journal.pone.0093834)
Supplement: Table S2 — Markers excluded from initial map construction because of transmission ratio distortion (TRD; co-dominant markers having <15% of homozygotes and dominant markers being present/absent in <15% or >40% individuals of the mapping population were omitted. Information on likely placement in the Draba nivalis genome, association with QTL traits, proportion of TRD, and mean fertility of the F2 individuals associated with the particular marker. (DOCX) [file pone.0093834.s002.docx]

**Supporting Information**

Table S2. Markers excluded from initial map construction because of transmission ratio distortion (TRD; co-dominant markers having <15% of homozygotes and dominant markers being present/absent in <15% or >40% individuals of the mapping population were omitted. Information on likely placement in the *Draba nivalis* genome, association with QTL traits, proportion of TRD, and mean fertility of the F_2_ individuals associated with the particular marker.

| \| Marker \| LG \| Position  (between marker A and B) \| \| QTL close \| TRD \| Trait \| Mean homozygote for A \| Mean homozygote for B \| Mean A present \| Mean B present \| Mean heterozygote \| \| --- \| --- \| --- \| --- \| --- \| --- \| --- \| --- \| --- \| --- \| --- \| --- \| \| A \| B \|  \|  \|  \| \|  \|  \|  \|  \|  \|  \|  \|  \|  \|  \|  \|  \| \| AFLP1 \| 1 \| AFLP47 \| A9 \| Seed set \| 146:211 \| Pollen fertility \| 55.62 \| - \| - \| 53.18 \| - \| \|  \|  \|  \|  \|  \|  \| Seed set \| 9.18 \| - \| - \| 7.90 \| - \| \|  \|  \|  \|  \|  \|  \| Flowering time \| 18.96 \| - \| - \| 18.24 \| - \| \|  \|  \|  \|  \|  \|  \| Number of flowers \| 13.89 \| - \| - \| 14.40 \| - \| \| AFLP2 \| Unplaced \|  \|  \|  \| 7:350 \| Pollen fertility \| 39.43 \| - \| - \| 54.47 \| - \| \|  \|  \|  \|  \|  \|  \| Seed set \| 8.29 \| - \| - \| 8.41 \| - \| \|  \|  \|  \|  \|  \|  \| Flowering time \| 18.00 \| - \| - \| 18.55 \| - \| \|  \|  \|  \|  \|  \|  \| Number of flowers \| 27.14 \| - \| - \| 13.93 \| - \| \| AFLP6 \| Unplaced \|  \|  \|  \| 4:353 \| Pollen fertility \| - \| 49.58 \| 54.22 \| - \| - \| \|  \|  \|  \|  \|  \|  \| Seed set \| - \| 5.00 \| 8.45 \| - \| - \| \|  \|  \|  \|  \|  \|  \| Flowering time \| - \| 17.25 \| 18.55 \| - \| - \| \|  \|  \|  \|  \|  \|  \| Number of flowers \| - \| 14.50 \| 14.19 \| - \| - \| \| AFLP14 \| 2 \| A112 \| A214 \| Seed set \| 42:316 \| Pollen fertility \| - \| 55.51 \| 54.08 \| - \| - \| \|  \|  \|  \|  \|  \|  \| Seed set \| - \| 8.83 \| 8.35 \| - \| - \| \|  \|  \|  \|  \|  \|  \| Flowering time \| - \| 19.88 \| 18.32 \| - \| - \| \|  \|  \|  \|  \|  \|  \| Number of flowers \| - \| 10.33 \| 14.72 \| - \| - \| \| AFLP37 \| Unplaced \|  \|  \|  \| 7:348 \| Pollen fertility \| 52.41 \| - \| - \| 54.27 \| - \| \|  \|  \|  \|  \|  \|  \| Seed set \| 8.71 \| - \| - \| 8.41 \| - \| \|  \|  \|  \|  \|  \|  \| Flowering time \| 21.86 \| - \| - \| 18.44 \| - \| \|  \|  \|  \|  \|  \|  \| Number of flowers \| 9.43 \| - \| - \| 14.33 \| - \| \| AFLP43 \| 8 \| AFLP64 \| AFLP39 \| - \| 43:310 \| Pollen fertility \| - \| 63.69 \| 53.07 \| - \| - \| \|  \|  \|  \|  \|  \|  \| Seed set \| - \| 9.20 \| 8.29 \| - \| - \| \|  \|  \|  \|  \|  \|  \| Flowering time \| - \| 18.24 \| 18.57 \| - \| - \| \|  \|  \|  \|  \|  \|  \| Number of flowers \| - \| 14.66 \| 14.22 \| - \| - \| \| AFLP48 \| Unplaced \|  \|  \|  \| 42:315 \| Pollen fertility \| 53.93 \| - \| - \| 54.23 \| - \| \|  \|  \|  \|  \|  \|  \| Seed set \| 8.59 \| - \| - \| 8.38 \| - \| \|  \|  \|  \|  \|  \|  \| Flowering time \| 18.36 \| - \| - \| 18.53 \| - \| \|  \|  \|  \|  \|  \|  \| Number of flowers \| 20.76 \| - \| - \| 13.28 \| - \| \| AFLP49 \| 8 \| AFLP73 \| AFLP64 \| - \| 45:312 \| Pollen fertility \| - \| 52.90 \| 54.35 \| - \| - \| \|  \|  \|  \|  \|  \|  \| Seed set \| - \| 7.88 \| 8.47 \| - \| - \| \|  \|  \|  \|  \|  \|  \| Flowering time \| - \| 18.53 \| 18.53 \| - \| - \| \|  \|  \|  \|  \|  \|  \| Number of flowers \| - \| 13.35 \| 14.30 \| - \| - \| \| AFLP55 \| 3 \| AFLP23 \| D11 \| Pollen/  Number of flowers \| 51:305 \| Pollen fertility \| 42.88 \| - \| - \| 56.00 \| - \| \|  \|  \|  \|  \|  \| Seed set \| 8.11 \| - \| - \| 8.42 \| - \| \|  \|  \|  \|  \|  \| Flowering time \| 19.53 \| - \| - \| 18.35 \| - \| \|  \|  \|  \|  \|  \|  \| Number of flowers \| 10.06 \| - \| - \| 14.75 \| - \| \| AFLP58 \| 8 \| AFLP67 \| AFLP73 \| - \| 47:309 \| Pollen fertility \| - \| 55.08 \| 54.00 \| - \| - \| \|  \|  \|  \|  \|  \|  \| Seed set \| - \| 8.25 \| 8.40 \| - \| - \| \|  \|  \|  \|  \|  \|  \| Flowering time \| - \| 17.98 \| 18.60 \| - \| - \| \|  \|  \|  \|  \|  \|  \| Number of flowers \| - \| 12.56 \| 14.31 \| - \| - \| \| AFLP59 \| 3 \| 0 \| AFLP23 \| Number of flowers \| 162:195 \| Pollen fertility \| - \| 62.74 \| 46.83 \| - \| - \| \|  \|  \|  \|  \|  \| Seed set \| - \| 8.56 \| 8.23 \| - \| - \| \|  \|  \|  \|  \|  \| Flowering time \| - \| 18.14 \| 18.84 \| - \| - \| \|  \|  \|  \|  \|  \|  \| Number of flowers \| - \| 14.51 \| 13.74 \| - \| - \| \| AFLP68 \| Unplaced \|  \|  \|  \| 0:158 \| Pollen fertility \| - \| - \| 54.08 \| - \| - \| \|  \|  \|  \|  \|  \|  \| Seed set \| - \| - \| 8.40 \| - \| - \| \|  \|  \|  \|  \|  \|  \| Flowering time \| - \| - \| 18.56 \| - \| - \| \|  \|  \|  \|  \|  \|  \| Number of flowers \| - \| - \| 14.22 \| - \| - \| \| AFLP70 \| 3 \| AFLP23 \| D11 \| Pollen/  Number of flowers \| 51:307 \| Pollen fertility \| 42.88 \| - \| - \| 55.91 \| - \| \|  \|  \|  \|  \|  \| Seed set \| 8.11 \| - \| - \| 8.45 \| - \| \|  \|  \|  \|  \|  \| Flowering time \| 19.53 \| - \| - \| 18.40 \| - \| \|  \|  \|  \|  \|  \|  \| Number of flowers \| 10.06 \| - \| - \| 14.90 \| - \| \| AFLP74 \| 3 \| 0 \| AFLP23 \| Number of flowers \| 165:193 \| Pollen fertility \| - \| 62.59 \| 46.67 \| - \| - \| \|  \|  \|  \|  \|  \| Seed set \| - \| 8.63 \| 8.21 \| - \| - \| \|  \|  \|  \|  \|  \|  \| Flowering time \| - \| 18.21 \| 18.86 \| - \| - \| \|  \|  \|  \|  \|  \|  \| Number of flowers \| - \| 14.75 \| 13.76 \| - \| - \| \| AFLP75 \| 8 \| B240 \| AFLP67 \| - \| 41:314 \| Pollen fertility \| - \| 51.62 \| 54.43 \| - \| - \| \|  \|  \|  \|  \|  \|  \| Seed set \| - \| 8.33 \| 8.40 \| - \| - \| \|  \|  \|  \|  \|  \|  \| Flowering time \| - \| 17.56 \| 18.69 \| - \| - \| \|  \|  \|  \|  \|  \|  \| Number of flowers \| - \| 13.68 \| 14.35 \| - \| - \| \| AFLP83 \| 1 \| AFLP65 \| A222 \| Seed set \| 141:205 \| Pollen fertility \| 54.50 \| - \| - \| 53.64 \| - \| \|  \|  \|  \|  \|  \|  \| Seed set \| 9.15 \| - \| - \| 7.93 \| - \| \|  \|  \|  \|  \|  \|  \| Flowering time \| 18.88 \| - \| - \| 18.25 \| - \| \|  \|  \|  \|  \|  \|  \| Number of flowers \| 14.27 \| - \| - \| 14.26 \| - \| \| AtC10_8 \| 8 \| AFLP67 \| AFLP73 \| - \| 37:317 \| Pollen fertility \| - \| 66.84 \| 52.85 \| - \| - \| \|  \|  \|  \|  \|  \|  \| Seed set \| - \| 9.20 \| 8.26 \| - \| - \| \|  \|  \|  \|  \|  \|  \| Flowering time \| - \| 17.41 \| 18.61 \| - \| - \| \|  \|  \|  \|  \|  \|  \| Number of flowers \| - \| 16.03 \| 14.06 \| - \| - \| \| SB2_15 \| 1 \| TRIM-Br_10 \| TRIM-Br_11 \|  \| 46:290 \| Pollen fertility \| - \| 58.30 \| 53.35 \| - \| - \| \|  \|  \|  \|  \|  \|  \| Seed set \| - \| 7.88 \| 8.43 \| - \| - \| \|  \|  \|  \|  \|  \|  \| Flowering time \| - \| 18.70 \| 18.45 \| - \| - \| \|  \|  \|  \|  \|  \|  \| Number of flowers \| - \| 13.91 \| 14.48 \| - \| - \| \| AtC10_20 \| Unplaced \|  \|  \|  \| 32:302 \| Pollen fertility \| 48.69 \| - \| - \| 54.60 \| - \| \|  \|  \|  \|  \|  \|  \| Seed set \| 8.41 \| - \| - \| 8.49 \| - \| \|  \|  \|  \|  \|  \|  \| Flowering time \| 19.53 \| - \| - \| 18.33 \| - \| \|  \|  \|  \|  \|  \|  \| Number of flowers \| 15.50 \| - \| - \| 14.40 \| - \| \| B116 \| 3 \| B228 \| AFLP35 \| - \| 170:154:30 \| Pollen fertility \| 55.92 \| 61.08 \| - \| - \| 51.56 \| \|  \|  \|  \|  \|  \|  \| Seed set \| 8.92 \| 8.50 \| - \| - \| 7.76 \| \|  \|  \|  \|  \|  \|  \| Flowering time \| 18.75 \| 17.24 \| - \| - \| 18.47 \| \|  \|  \|  \|  \|  \|  \| Number of flowers \| 14.69 \| 14.25 \| - \| - \| 13.88 \| \| A115 \| 8 \| B234 \| B240 \| - \| 137:176:43 \| Pollen fertility \| 58.61 \| 52.31 \| - \| - \| 51.32 \| \|  \|  \|  \|  \|  \|  \| Seed set \| 8.98 \| 8.00 \| - \| - \| 8.02 \| \|  \|  \|  \|  \|  \|  \| Flowering time \| 18.77 \| 17.59 \| - \| - \| 18.57 \| \|  \|  \|  \|  \|  \|  \| Number of flowers \| 17.44 \| 13.38 \| - \| - \| 12.00 \| \| A138 \| 8 \| B234 \| B240 \| - \| 135:178:43 \| Pollen fertility \| 58.98 \| 52.09 \| - \| - \| 51.48 \| \|  \|  \|  \|  \|  \|  \| Seed set \| 9.06 \| 8.00 \| - \| - \| 8.04 \| \|  \|  \|  \|  \|  \|  \| Flowering time \| 18.67 \| 17.66 \| - \| - \| 18.57 \| \|  \|  \|  \|  \|  \|  \| Number of flowers \| 17.73 \| 13.36 \| - \| - \| 11.93 \| \| B140 \| 8 \| AFLP34 \| B234 \| - \| 139:163:49 \| Pollen fertility \| 58.71 \| 52.08 \| - \| - \| 50.77 \| \|  \|  \|  \|  \|  \|  \| Seed set \| 9.20 \| 8.22 \| - \| - \| 7.75 \| \|  \|  \|  \|  \|  \|  \| Flowering time \| 18.53 \| 18.22 \| - \| - \| 18.65 \| \|  \|  \|  \|  \|  \|  \| Number of flowers \| 14.85 \| 13.81 \| - \| - \| 13.52 \| \| B10 \| 8 \| B234 \| B240 \| - \| 123:191:45 \| Pollen fertility \| 58.50 \| 52.64 \| - \| - \| 51.75 \| \|  \|  \|  \|  \|  \|  \| Seed set \| 8.90 \| 8.41 \| - \| - \| 8.07 \| \|  \|  \|  \|  \|  \|  \| Flowering time \| 18.53 \| 17.38 \| - \| - \| 18.75 \| \|  \|  \|  \|  \|  \|  \| Number of flowers \| 17.73 \| 13.91 \| - \| - \| 11.97 \| \| B103 \| 8 \| B240 \| AFLP73 \| - \| 116:185:39 \| Pollen fertility \| 58.12 \| 57.92 \| - \| - \| 51.52 \| \|  \|  \|  \|  \|  \|  \| Seed set \| 8.94 \| 7.69 \| - \| - \| 8.26 \| \|  \|  \|  \|  \|  \|  \| Flowering time \| 18.49 \| 18.08 \| - \| - \| 18.44 \| \|  \|  \|  \|  \|  \|  \| Number of flowers \| 16.13 \| 12.43 \| - \| - \| 13.67 \| \| B207 \| 8 \| AFLP67 \| AFLP73 \| - \| 132:165:48 \| Pollen fertility \| 56.29 \| 53.96 \| - \| - \| 52.52 \| \|  \|  \|  \|  \|  \|  \| Seed set \| 8.41 \| 8.27 \| - \| - \| 8.43 \| \|  \|  \|  \|  \|  \|  \| Flowering time \| 19.04 \| 17.93 \| - \| - \| 18.18 \| \|  \|  \|  \|  \|  \|  \| Number of flowers \| 16.75 \| 13.00 \| - \| - \| 13.13 \| \| B233 \| Unplaced \|  \|  \|  \| 29:156:117 \| Pollen fertility \| 64.95 \| 54.11 \| - \| - \| 52.14 \| \|  \|  \|  \|  \|  \|  \| Seed set \| 9.25 \| 8.52 \| - \| - \| 8.03 \| \|  \|  \|  \|  \|  \|  \| Flowering time \| 17.37 \| 17.88 \| - \| - \| 19.34 \| \|  \|  \|  \|  \|  \|  \| Number of flowers \| 16.04 \| 15.90 \| - \| - \| 12.42 \| \| A218 \| 8 \| B234 \| B240 \| - \| 96:143:39 \| Pollen fertility \| 58.15 \| 52.09 \| - \| - \| 51.57 \| \|  \|  \|  \|  \|  \|  \| Seed set \| 9.27 \| 8.05 \| - \| - \| 8.35 \| \|  \|  \|  \|  \|  \|  \| Flowering time \| 18.38 \| 17.16 \| - \| - \| 18.46 \| \|  \|  \|  \|  \|  \|  \| Number of flowers \| 19.26 \| 15.39 \| - \| - \| 13.36 \| \| D2 \| 3 \| B228 \| AFLP35 \| - \| 173:158:22 \| Pollen fertility \| 54.57 \| 64.93 \| - \| - \| 51.79 \| \|  \|  \|  \|  \|  \|  \| Seed set \| 8.93 \| 9.20 \| - \| - \| 7.65 \| \|  \|  \|  \|  \|  \|  \| Flowering time \| 18.43 \| 17.81 \| - \| - \| 18.72 \| \|  \|  \|  \|  \|  \|  \| Number of flowers \| 13.95 \| 10.67 \| - \| - \| 14.71 \| \|  \|  \|  \|  \|  \|  \|  \|  \|  \|  \|  \|  \| \|  \|  \|  \|  \|  \|  \|  \|  \|  \|  \|  \|  \| \|  \|  \|  \|  \|  \|  \|  \|  \|  \|  \|  \|  \| \|  \|  \|  \|  \|  \|  \|  \|  \|  \|  \|  \|  \| |
| --- | --- | --- | --- | --- | --- | --- | --- | --- | --- | --- | --- | --- | --- | --- | --- | --- | --- | --- | --- | --- | --- | --- | --- | --- | --- | --- | --- | --- | --- | --- | --- | --- | --- | --- | --- | --- | --- | --- | --- | --- | --- | --- | --- | --- | --- | --- | --- | --- | --- | --- | --- | --- | --- | --- | --- | --- | --- | --- | --- | --- | --- | --- | --- | --- | --- | --- | --- | --- | --- | --- | --- | --- | --- | --- | --- | --- | --- | --- | --- | --- | --- | --- | --- | --- | --- | --- | --- | --- | --- | --- | --- | --- | --- | --- | --- | --- | --- | --- | --- | --- | --- | --- | --- | --- | --- | --- | --- | --- | --- | --- | --- | --- | --- | --- | --- | --- | --- | --- | --- | --- | --- | --- | --- | --- | --- | --- | --- | --- | --- | --- | --- | --- | --- | --- | --- | --- | --- | --- | --- | --- | --- | --- | --- | --- | --- | --- | --- | --- | --- | --- | --- | --- | --- | --- | --- | --- | --- | --- | --- | --- | --- | --- | --- | --- | --- | --- | --- | --- | --- | --- | --- | --- | --- | --- | --- | --- | --- | --- | --- | --- | --- | --- | --- | --- | --- | --- | --- | --- | --- | --- | --- | --- | --- | --- | --- | --- | --- | --- | --- | --- | --- | --- | --- | --- | --- | --- | --- | --- | --- | --- | --- | --- | --- | --- | --- | --- | --- | --- | --- | --- | --- | --- | --- | --- | --- | --- | --- | --- | --- | --- | --- | --- | --- | --- | --- | --- | --- | --- | --- | --- | --- | --- | --- | --- | --- | --- | --- | --- | --- | --- | --- | --- | --- | --- | --- | --- | --- | --- | --- | --- | --- | --- | --- | --- | --- | --- | --- | --- | --- | --- | --- | --- | --- | --- | --- | --- | --- | --- | --- | --- | --- | --- | --- | --- | --- | --- | --- | --- | --- | --- | --- | --- | --- | --- | --- | --- | --- | --- | --- | --- | --- | --- | --- | --- | --- | --- | --- | --- | --- | --- | --- | --- | --- | --- | --- | --- | --- | --- | --- | --- | --- | --- | --- | --- | --- | --- | --- | --- | --- | --- | --- | --- | --- | --- | --- | --- | --- | --- | --- | --- | --- | --- | --- | --- | --- | --- | --- | --- | --- | --- | --- | --- | --- | --- | --- | --- | --- | --- | --- | --- | --- | --- | --- | --- | --- | --- | --- | --- | --- | --- | --- | --- | --- | --- | --- | --- | --- | --- | --- | --- | --- | --- | --- | --- | --- | --- | --- | --- | --- | --- | --- | --- | --- | --- | --- | --- | --- | --- | --- | --- | --- | --- | --- | --- | --- | --- | --- | --- | --- | --- | --- | --- | --- | --- | --- | --- | --- | --- | --- | --- | --- | --- | --- | --- | --- | --- | --- | --- | --- | --- | --- | --- | --- | --- | --- | --- | --- | --- | --- | --- | --- | --- | --- | --- | --- | --- | --- | --- | --- | --- | --- | --- | --- | --- | --- | --- | --- | --- | --- | --- | --- | --- | --- | --- | --- | --- | --- | --- | --- | --- | --- | --- | --- | --- | --- | --- | --- | --- | --- | --- | --- | --- | --- | --- | --- | --- | --- | --- | --- | --- | --- | --- | --- | --- | --- | --- | --- | --- | --- | --- | --- | --- | --- | --- | --- | --- | --- | --- | --- | --- | --- | --- | --- | --- | --- | --- | --- | --- | --- | --- | --- | --- | --- | --- | --- | --- | --- | --- | --- | --- | --- | --- | --- | --- | --- | --- | --- | --- | --- | --- | --- | --- | --- | --- | --- | --- | --- | --- | --- | --- | --- | --- | --- | --- | --- | --- | --- | --- | --- | --- | --- | --- | --- | --- | --- | --- | --- | --- | --- | --- | --- | --- | --- | --- | --- | --- | --- | --- | --- | --- | --- | --- | --- | --- | --- | --- | --- | --- | --- | --- | --- | --- | --- | --- | --- | --- | --- | --- | --- | --- | --- | --- | --- | --- | --- | --- | --- | --- | --- | --- | --- | --- | --- | --- | --- | --- | --- | --- | --- | --- | --- | --- | --- | --- | --- | --- | --- | --- | --- | --- | --- | --- | --- | --- | --- | --- | --- | --- | --- | --- | --- | --- | --- | --- | --- | --- | --- | --- | --- | --- | --- | --- | --- | --- | --- | --- | --- | --- | --- | --- | --- | --- | --- | --- | --- | --- | --- | --- | --- | --- | --- | --- | --- | --- | --- | --- | --- | --- | --- | --- | --- | --- | --- | --- | --- | --- | --- | --- | --- | --- | --- | --- | --- | --- | --- | --- | --- | --- | --- | --- | --- | --- | --- | --- | --- | --- | --- | --- | --- | --- | --- | --- | --- | --- | --- | --- | --- | --- | --- | --- | --- | --- | --- | --- | --- | --- | --- | --- | --- | --- | --- | --- | --- | --- | --- | --- | --- | --- | --- | --- | --- | --- | --- | --- | --- | --- | --- | --- | --- | --- | --- | --- | --- | --- | --- | --- | --- | --- | --- | --- | --- | --- | --- | --- | --- | --- | --- | --- | --- | --- | --- | --- | --- | --- | --- | --- | --- | --- | --- | --- | --- | --- | --- | --- | --- | --- | --- | --- | --- | --- | --- | --- | --- | --- | --- | --- | --- | --- | --- | --- | --- | --- | --- | --- | --- | --- | --- | --- | --- | --- | --- | --- | --- | --- | --- | --- | --- | --- | --- | --- | --- | --- | --- | --- | --- | --- | --- | --- | --- | --- | --- | --- | --- | --- | --- | --- | --- | --- | --- | --- | --- | --- | --- | --- | --- | --- | --- | --- | --- | --- | --- | --- | --- | --- | --- | --- | --- | --- | --- | --- | --- | --- | --- | --- | --- | --- | --- | --- | --- | --- | --- | --- | --- | --- | --- | --- | --- | --- | --- | --- | --- | --- | --- | --- | --- | --- | --- | --- | --- | --- | --- | --- | --- | --- | --- | --- | --- | --- | --- | --- | --- | --- | --- | --- | --- | --- | --- | --- | --- | --- | --- | --- | --- | --- | --- | --- | --- | --- | --- | --- | --- | --- | --- | --- | --- | --- | --- | --- | --- | --- | --- | --- | --- | --- | --- | --- | --- | --- | --- | --- | --- | --- | --- | --- | --- | --- | --- | --- | --- | --- | --- | --- | --- | --- | --- | --- | --- | --- | --- | --- | --- | --- | --- | --- | --- | --- | --- | --- | --- | --- | --- | --- | --- | --- | --- | --- | --- | --- | --- | --- | --- | --- | --- | --- | --- | --- | --- | --- | --- | --- | --- | --- | --- | --- | --- | --- | --- | --- | --- | --- | --- | --- | --- | --- | --- | --- | --- | --- | --- | --- | --- | --- | --- | --- | --- | --- | --- | --- | --- | --- | --- | --- | --- | --- | --- | --- | --- | --- | --- | --- | --- | --- | --- | --- | --- | --- | --- | --- | --- | --- | --- | --- | --- | --- | --- | --- | --- | --- | --- | --- | --- | --- | --- | --- | --- | --- | --- | --- | --- | --- | --- | --- | --- | --- | --- | --- | --- | --- | --- | --- | --- | --- | --- | --- | --- | --- | --- | --- | --- | --- | --- | --- | --- | --- | --- | --- | --- | --- | --- | --- | --- | --- | --- | --- | --- | --- | --- | --- | --- | --- | --- | --- | --- | --- | --- | --- | --- | --- | --- | --- | --- | --- | --- | --- | --- | --- | --- | --- | --- | --- | --- | --- | --- | --- | --- | --- | --- | --- | --- | --- | --- | --- | --- | --- | --- | --- | --- | --- | --- | --- | --- | --- | --- | --- | --- | --- | --- | --- | --- | --- | --- | --- | --- | --- | --- | --- | --- | --- | --- | --- | --- | --- | --- | --- | --- | --- | --- | --- | --- | --- | --- | --- | --- | --- | --- | --- | --- | --- | --- | --- | --- | --- | --- | --- | --- | --- | --- | --- | --- | --- | --- | --- | --- | --- | --- | --- | --- | --- | --- | --- | --- | --- | --- | --- | --- | --- | --- | --- | --- | --- | --- | --- | --- | --- | --- | --- | --- | --- | --- | --- | --- | --- | --- | --- | --- | --- | --- | --- | --- | --- | --- | --- | --- | --- | --- | --- | --- | --- | --- | --- | --- | --- | --- | --- | --- | --- | --- | --- | --- | --- | --- | --- | --- | --- | --- | --- | --- | --- | --- | --- | --- | --- | --- | --- | --- | --- | --- | --- | --- | --- | --- | --- | --- | --- | --- | --- | --- | --- | --- | --- | --- | --- | --- | --- | --- | --- | --- | --- | --- | --- | --- | --- | --- | --- | --- | --- | --- | --- | --- | --- | --- | --- | --- | --- | --- | --- | --- | --- | --- | --- | --- | --- | --- | --- | --- | --- | --- | --- | --- | --- | --- | --- | --- | --- | --- | --- | --- | --- | --- | --- | --- | --- | --- | --- | --- | --- | --- | --- | --- | --- | --- | --- | --- | --- | --- | --- | --- | --- | --- | --- | --- | --- | --- | --- | --- | --- | --- | --- | --- | --- | --- | --- | --- | --- | --- | --- | --- | --- | --- | --- | --- | --- | --- | --- | --- | --- | --- | --- | --- | --- | --- | --- | --- | --- | --- | --- | --- | --- | --- | --- | --- | --- | --- | --- | --- | --- | --- | --- | --- | --- | --- | --- | --- | --- | --- | --- | --- | --- | --- | --- | --- | --- | --- | --- | --- | --- | --- | --- | --- | --- | --- | --- | --- | --- | --- | --- | --- | --- | --- | --- | --- | --- | --- | --- | --- | --- | --- | --- | --- | --- | --- | --- | --- | --- | --- | --- | --- | --- | --- | --- | --- | --- | --- | --- | --- | --- | --- | --- | --- | --- | --- | --- |
